# Evaluation of the Efficacy of Platelet-Rich Plasma Injections With and Without Microneedling for Managing Atrophic Facial Acne Scars: A Prospective Comparative Study

**DOI:** 10.7759/cureus.60957

**Published:** 2024-05-23

**Authors:** Manishaa V, Senthil Murugan P

**Affiliations:** 1 Oral and Maxillofacial Surgery, Saveetha Dental College and Hospitals, Saveetha Institute of Medical and Technical Sciences, Saveetha University, Chennai, IND

**Keywords:** scarring, esthetics, microneedling, platelet rich plasma, acne

## Abstract

Background and aim

The majority of acne has the potential to transform into facial scars, which have a physical and psychological effect on the individual. There are plenty of treatment options to manage such scars. The aim of this study is to assess the comparative effect of the injection of platelet-rich plasma (PRP) alone, with that of the injection of PRP with microneedling, in the reduction of atrophic facial acne scars.

Methods

A total of 30 participants were included in this study, divided into two groups (n = 15). Patients in Group I received intradermal injection of PRP only, and Group II included patients receiving intradermal injection of PRP with microneedling. The scar appearance was evaluated at baseline, after one, two, and three months using Goodman Baron’s scar scale. The statistics were analysed using the Chi-square and Student's t-tests.

Results

Patients in the PRP with microneedling group had lower acne scar scores on the Goodman Baron scale compared to those who received only PRP. The acne scores were statistically significant (p-value < 0.05) in the second and third months of treatment in Group II.

Conclusion

The addition of microneedling to PRP has proven to be effective in the reduction of facial acne scars. However, different types of scars require different modalities of treatment, and the final decision lies in the hands of the operator and the requirements of the patients.

## Introduction

Acne vulgaris is the most common dermatological problem prevalent among people worldwide, transcending age, gender, and ethnicity. It has a varied clinical presentation, ranging from pustules to comedones [1]. While it is often associated with adolescence, acne can persist into adulthood and even emerge for the first time later in life [2]. Factors such as genetics, hormones, bacteria, and inflammation contribute to its development [3]. 

Adult acne can be of three types: atrophic, keloid, and hypertrophic. Atrophic scars are classified into ice packs, rolling, and boxcars. Acne forms due to obstruction of the hair follicles with sebum and dead skin cells, creating an ideal environment for bacteria to thrive. A variety of reasons can cause clogging of these pores that can result in acne. Several risk factors can trigger acne such as hormonal changes like pregnancy and puberty, medical conditions like polycystic ovarian syndrome (PCOS), cigarette smoking, stress, applications of oil-based products, and a history of acne. Understanding the causes, treatment options and prevention strategies for facial acne is crucial for effectively managing this condition and maintaining healthy skin [4]. Fortunately, several topical and systemic treatment options are available to help manage facial acne and prevent future breakouts. These include topical medications containing ingredients like beta hydroxy acids such as salicylic acid, retinoids, bactericidal agents like benzoyl peroxide, and antibiotics that can aid in opening up the pores, thereby decreasing inflammation and killing the acne-causing bacteria [5]. Dermatological procedures like chemical peels, microdermabrasion, microneedling, low-level lasers, and corticosteroid injections can be helpful in managing stubborn acne lesions, reducing inflammation, and preventing scarring [6]. Systemic management includes administration of antibiotics, hormonal therapies, and isotretinoin.

Platelet-rich plasma (PRP) is a type of regenerative medical treatment that utilizes patients’ own growth factors obtained from the patient's own blood. The resulting plasma, rich in platelets, is then injected back into the patient at the site of injury or treatment [7]. PRP is used in various medical fields, including tendon injuries, osteoarthritis, and skin rejuvenation [8]. It has found increasing importance in the management of facial acne in recent times due to its wound-healing capabilities. Microneedling, or needle dermabrasion, is one of the least invasive dermatological procedures. It involves the use of a device with multiple small needles that induce micro-trauma to the dermis [9]. Microneedling is commonly used to address various dermatological conditions such as post-acne scars, wrinkles, surface irregularities, enlarged pores, and stretch marks, and it helps produce an even skin tone [10]. 

The purpose of the present study is to evaluate and compare the effects of injection of PRP with or without microneedling in the reduction of facial atrophic acne scars, measured using the Goodman Baron’s scar scale. The objective of the study is to analyse the reduction of facial scarring and evaluate the effect of PRP and microneedling on the improvement of scarring and skin pigmentation due to acne. 

## Materials and methods

Study setting

The study included patients who reported to the Oral and Maxillofacial Surgery Department with facial acne seeking treatment for the same between January 2023 and September 2023. The study was approved by the Saveetha Institutional Ethics Committee (IHEC/SDC/OMFS-2104/22/326) at Saveetha Dental College and Hospitals, Saveetha Institute of Medical and Technical Sciences, Saveetha University, Chennai, India. The patient was explained about the study and informed consent was obtained from them.

Inclusion criteria

Participants included those from 20 to 40 years of age who were suffering from an atrophic type of acne scar with no signs of active infection.

Exclusion criteria

Patients presenting with systemic diseases such as anaemia, coagulation defects, blood dyscrasias, and herpes simplex; patients taking anticoagulants or immunosuppressive drugs; patients with active acne lesions; and pregnant and lactating women were excluded. Patients with irregular follow-up or those who were lost to follow-up were excluded.

Intervention 

Thirty patients with facial acne were accepted in the study. The participants were separated into two groups - Group I (n = 15) and Group II (n = 15). The patients were assigned into two groups randomly based on sealed opaque envelopes that were prepared by the primary investigator. The study was double-blinded, i.e., both the operator and the participant were unaware of the study grouping. Group I participants were treated with intradermal PRP injections, and Group II participants were treated with intradermal PRP injections and microneedling. 

**Figure 1:**
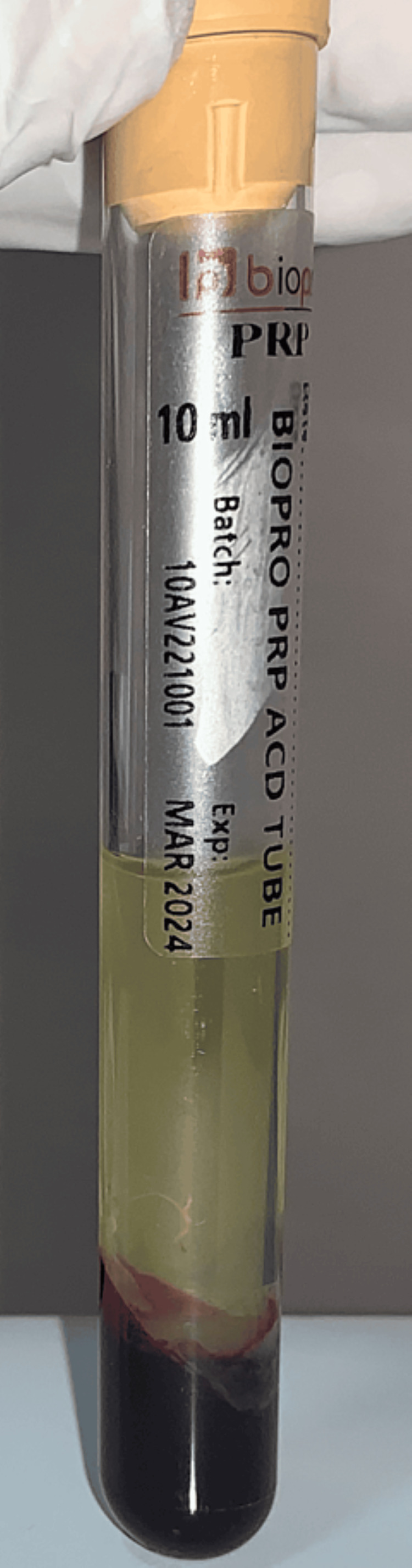
Preparation of platelet-rich plasma after the first spin of centrifugation showing distinct layers

**Figure 2:**
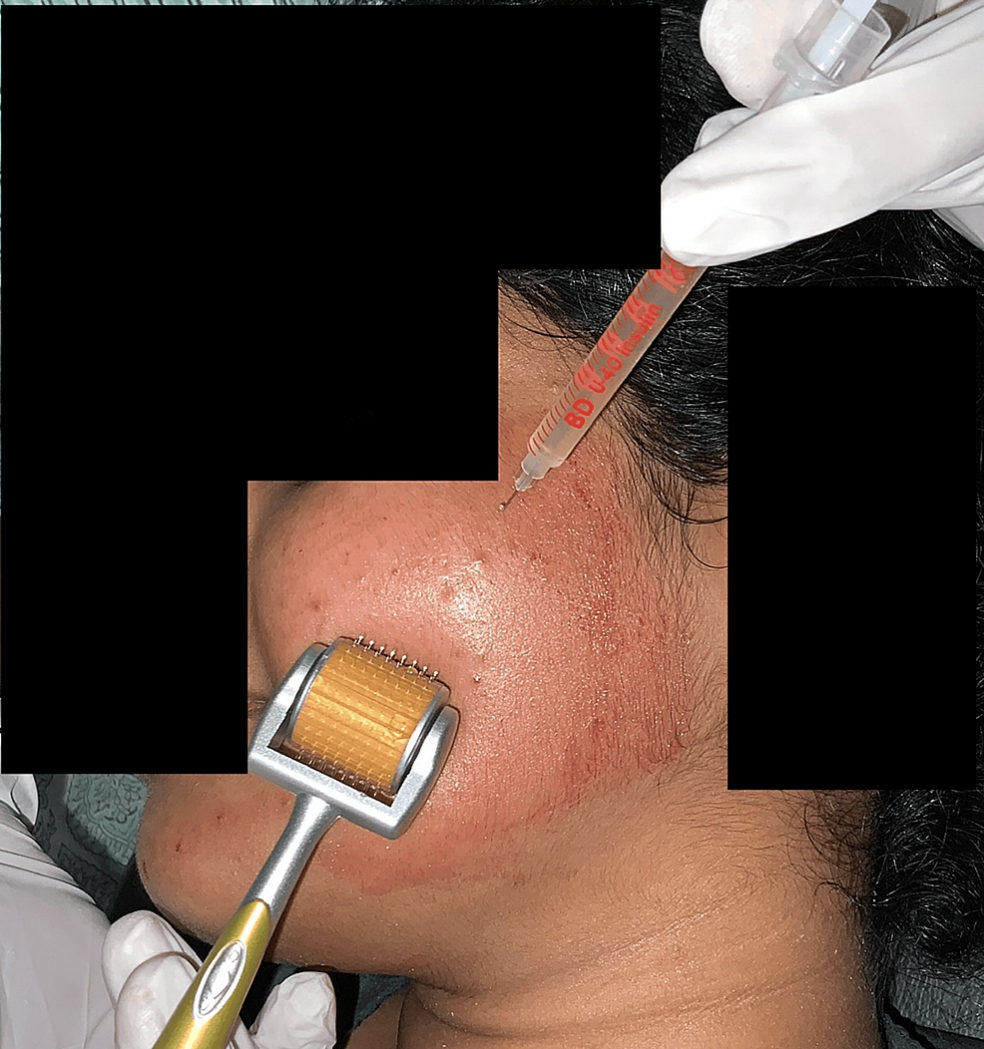
Microneedling done along with the injection of platelet-rich plasma

PRP was prepared for each of these participants using their blood. Initially, 10 ml of autologous blood was withdrawn from the participant and was subjected to a double-spin centrifugation in test tubes [8]. First, the blood was centrifuged for a period of 19 minutes, at a speed of 1500 revolutions per minute (rpm). This leads to the formation of a platelet-poor plasma, a buffy coat, and RBC sediments (Figure 1). The supernatant was subjected to centrifugation again at 3400 rpm for 10 minutes, thus obtaining PRP. Prior to PRP injections, topical anaesthetic gel was applied over the area of interest bilaterally. In Group I participants, PRP was injected intradermally around the acne scars using 30G (insulin syringes), until a small bleb or elevation was seen to confirm deposition of the PRP. In Group II participants, prior to the injection of PRP, a derma-roller (with 192 needles and of size 1.5 mm) was used in all four directions (Figure 2). The microneedling procedure was concluded with the appearance of pinpoint bleeding spots. The face was then cleaned with a sterile gauze and the patients were instructed to leave the surface untouched for one hour.

All participants were subjected to three sessions, with each session after a one-month break. Participants of both groups were advised to use sun protection cream every day. 

Assessment 

**Figure 3:**
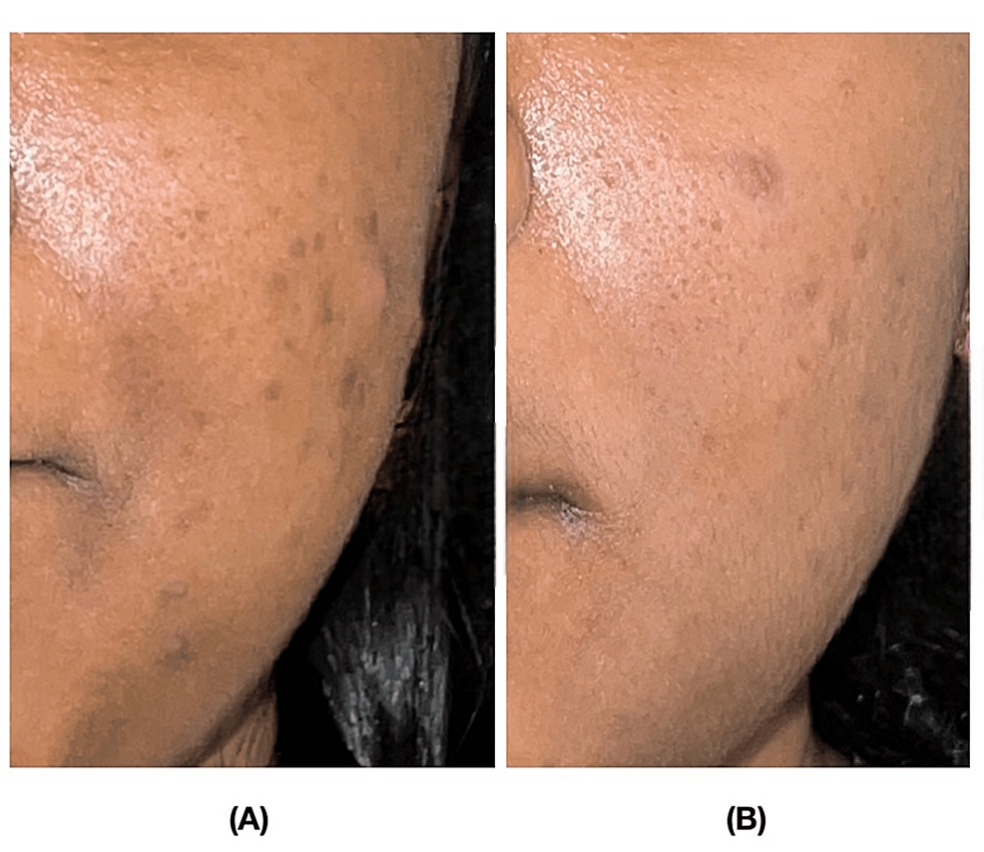
Comparison of the acne scores in a participant of Group I (PRP only) A) At baseline; B) At three months PRP: platelet-rich plasma

**Figure 4:**
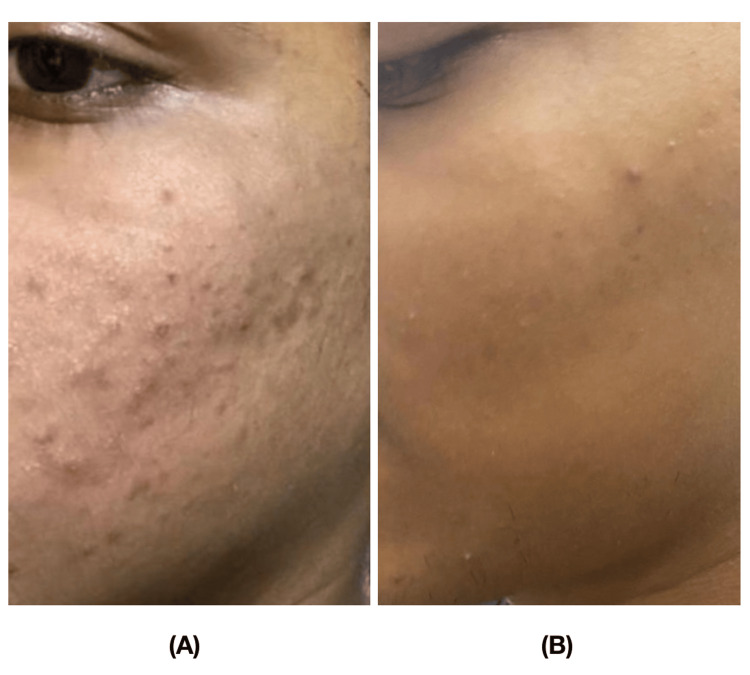
Comparison of the acne scores in a participant of Group II (PRP with microneedling) A) At baseline; B) At three months PRP: platelet-rich plasma

Photographs were taken prior to the start of treatment at all time intervals. The procedure was performed in three sessions with intervals of one month in between each session. The acne scars were evaluated in all participants bilaterally on the face prior to the end of the third session. The acne scars were quantitatively assessed using the Goodman Baron’s scale prior to each session, at zero, one, and two months, and one month after the last session for follow-up. The Goodman Baron’s scar scale assesses scars based on their grade or type, as well as the number of lesions (Table 1) (Figures 3-4).

**Table 1:** Goodman Baron’s Scar scale

Grade (Type)	Number of lesions (1-10)	Number of lesions (11-20)	Number of lesions (>20)
(A) Mild scarring (one point each) - Macular erythematous or pigmented, mildly atrophic dish-like	1 point	2 points	3 points
(B) Moderate scarring (two points each) - Moderately atrophic dish-like, punched out with shallow bases, small scars (<5 mm), shallow but broad atrophic scars	2 points	4 points	6 points
(C) Severe scarring (three points each) - Punched out with deep but normal bases, small scars (<5 mm), punched out with deep abnormal bases, small scars (<5 mm), linear or troughed dermal scarring, deep, broad atrophic areas	3 points	6 points	9 points
(D) Hyperplastic papular scars	4 points	8 points	6 points
(E) Hyperplastic keloidal, hypertrophic scars	Area <5 square cm - 6 points	Area 5-20 square cm - 1 point	Area >20 square cm - 18 points

Statistical analysis

Data were analysed using IBM SPSS Statistics for Windows, Version 23 (Released 2015; IBM Corp., Armonk, New York, United States). The categorical variables were compared using the Chi-square test. The intra-group analyses were done using the Wilcoxon signed ranks test before and after the treatment. The independent groups were compared using the Mann-Whitney U-test and Student’s t-test. A p-value of <0.05 was considered significant.

## Results

The study consists of two groups, with each group having 15 participants. The demographic data of patients has been tabulated (Table 2). The participants had a mean age of 27 ± 1.3 years in Group I and 27 ± 2.0 years in Group II. Amongst the 30 participants, there were 13 males and 17 females.

**Table 2:** Demographic data of participants

Demographic data	Group I	Group II
Age (years)	27 ± 1.3	25 ± 2.0
Gender (male, female)	8, 7	5, 10

The baseline values of acne scars as measured using the Goodman Baron’s scale were comparable in both groups and were not statistically significant (p-value > 0.05). The patients in Group I (PRP) showed a slight improvement in the scar reduction levels after the first session at the end of one month, while those in Group II (PRP with microneedling) showed comparatively better reduction in the scar scores. However, these values were statistically not significant (p-value = 0.878) (Table 3).

**Table 3:** Mean scar scores according to Goodman Baron’s scale among the participants of both groups PRP: platelet-rich plasma; * statistically significant Statistical analysis was done using the Chi-square test and Student's t-test

Mean scar scale scores	Group I - PRP	Group II - PRP with microneedling	p-value
Baseline	29.0 +/- 3.07	28.87 +/- 5.27	0.885
After one month	23.11 +/- 2.49	22.78 +/- 4.1	0.878
After two months	17.57 +/- 4.51	14.15 +/- 3.05	0.001*
After three months	10.09 +/- 3.57	7.09 +/- 1.46	0.001*

**Figure 5:**
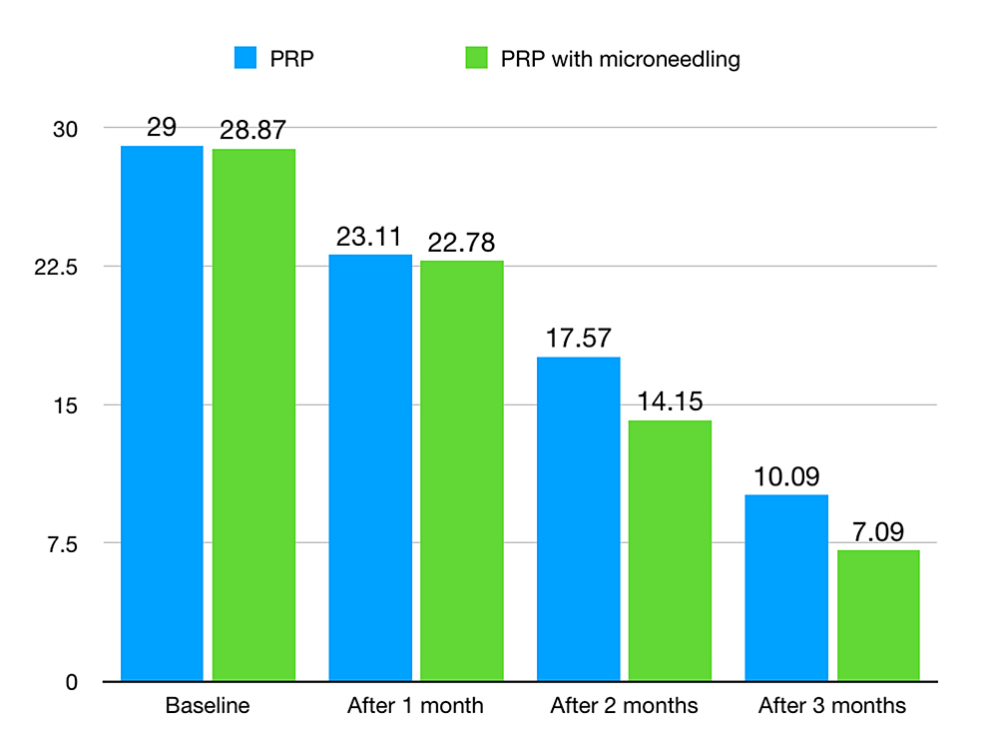
Graphical representation of the acne scar scores amongst participants of both groups PRP: platelet-rich plasma

Similarly, at the end of the second session, the scar values in Group II were lesser than those of Group I and were statistically significant (p-value < 0.001). Throughout the study, the scores were consistently lesser in Group II, thus proving that PRP with microneedling has better efficiency compared to PRP alone. At the end of the study in the third month, participants of Group II showed a statistically significant drop in the scar scores when compared to the participants of Group I (p-value < 0.001) (Figure 5). In this study, no complications or adverse effects were reported due to these drugs.

## Discussion

Although a frequent issue, scarring from facial acne is extremely challenging to treat, it occurs as a result of several reasons leading to inflammation. Atrophic acne scars can be treated using a variety of techniques, with varying degrees of success. Sebum is an oily product synthesised and secreted from the sebaceous glands located within the skin to produce an oily substance called sebum. Overproduction of sebum can lead to clogged pores and acne breakouts [11]. Another aetiology is a bacterium named *Propionibacterium acnes* (*P. acnes*) that resides on the skin and may cause the formation of acne. When *P. acnes* is entrapped within the blocked pores, it can multiply rapidly, causing inflammation, and the formation of pimples [12]. Hormonal fluctuations, particularly during puberty, menstruation, pregnancy, and menopause, can trigger acne flare-ups. Hence, the treatment strategies must focus on the elimination or reduction of the etiological factors. 

The introduction of PRP as a product of blood dates back to 1970, when it was used for the management of thrombocytopenia. Since then, it has been used in a variety of medical fields like sports medicine, paediatric surgery, plastic surgery, dermatology, and gynaecology [13]. It is currently used in dermatology as a part of regenerative medicine and in skin rejuvenation and alopecia. 

PRP therapy has been explored as a potential treatment for acne, particularly for acne scarring. When injected into areas affected by acne scars, PRP can stimulate collagen production and promote tissue regeneration, leading to smoother, healthier-looking skin [14]. PRP contains about 20 growth factors and other bioactive proteins that may help reduce inflammation and improve overall skin quality. Similarly, microneedling triggers the physiology of wound healing and the inflammatory mechanism, causing an increase in elastin and collagen formation by creating minute traumatic injuries to the dermis. This, in turn, leads to an improvement in the texture and turgidity of the skin, enhancing its overall appearance [15]. The micro punctures are created in the epidermis with a diameter of approximately four cells. This layer contains stem cells that are rich in collagen and growth factors such as platelet-derived growth factors, transforming growth factor-alpha and beta, fibroblast growth factor, and connective tissue growth factor. The activation of these growth factors initiates neovascularisation and neocollagenesis [16]. 

Fabbrocini et al. highlighted in their study the improvement in post-acne scars within five sittings in intervals of four to eight weeks with the application of PRP [17]. In another study conducted by Ismail et al., atrophic acne scars respond better to the treatment using microneedling and the application of PRP [18]. Contrary to the results of our study, a study conducted by Meghna Gupta et al. showed no added benefit with the topical application of PRP. The study evaluated the ECCA (Ecchelle D’Evaluation Cliniques des Cicatrices D’Acne) scores of two groups of patients with facial acne, one group consisting of patients treated with microneedling alone and another with a combination of microneedling with PRP [19]. 

A study was conducted by Porwal et al., comparing the Dermatology Life Quality Index between patients treated with microneedling for the management of acne scars, with or without PRP injections. These patients showed a betterment of 58.47% and 42.67% in the scar scores respectively at the end of the third session [20]. PRP with alternative sessions of microneedling have also been done to evaluate their effects on the reduction of post-acne scars. This study was undertaken by Ibrahim et al. with 90 patients who were distributed into three groups: PRP injection alone, microneedling alone, and alternating sessions of PRP and microneedling. Patients with alternating sessions showed superior results [21].

Several other modalities of treatment have also been researched alongside PRP to evaluate their effectiveness. Mumtaz et al. have evaluated the effects of PRP and trichloroacetic acid (TCA) for the management of atrophic acne scars. The results revealed better results with intra-dermal PRP when compared to TCA [22]. Ibrahim et al. conducted a similar study comparing the use of TCA (dot peeling), subcision, and PRP application [23]. 

Our study highlights the added advantage of microneedling with PRP injection that can be attributed to the improved penetration of the PRP due to the micro-injuries created. This study involves intradermal injection of PRP followed by microneedling, contrary to previous studies wherein microneedling was followed by topical application of PRP. The limitations of the present study include a small sample size and bias in the observer while evaluating the scores. The future scope of the study included performing the study in a larger sample size for generalising the results and comparing PRP with microneedling with other treatment options to provide patients with better results. The same treatment protocol can also be applied to evaluate their effect on different types of acne scars.

## Conclusions

In conclusion, both intradermal platelet rock plasma alone or in combination with microneedling yield good results. However, the combination of PRP with microneedling has proven to be more effective in the management of facial acne in this study. It improves both the aesthetic outcomes and the patient satisfaction. The added benefits of microneedling are attributed to the formation of micro-injuries that allow better penetration of the PRP and result in improved healing rates. Further research is required to investigate the effects of microneedling on the treatment of various types of atrophic facial acne scars. By understanding the underlying factors contributing to acne and implementing appropriate prevention and treatment strategies, one can effectively manage the condition.
